# The negative factors influencing the career intention of general practice trainees in eastern China: a qualitative study

**DOI:** 10.1186/s12909-022-03456-x

**Published:** 2022-05-21

**Authors:** Lei Tang, Huan Yang, Zhuxin Mao, Quan Li, Shunping Li

**Affiliations:** 1 Centre for Health Management and Policy Research, School of Public Health, Cheeloo College of Medicine, Shandong University, 250012 Jinan, China; 2 NHC Key Lab of Health Economics and Policy Research, Shandong University, 250012 Jinan, China; 3 Center for Health Preference Research, Shandong University, 250012 Jinan, China; 4 Department of Human Resources, The Fourth Affiliated Hospital, Zhejiang University School of Medicine, 322000 Yiwu, China; 5 School of Public Administration, Southwestern University of Finance and Economics, 611130 Chengdu, China; 6 Cheeloo College of Medicine, Shandong University, 250012 Jinan, China

**Keywords:** China, Career intention, Trainees, General practitioner, Qualitative study

## Abstract

**Background:**

There is an acute shortage of general practitioners (GPs) in China, and GP trainees seem to be less willing to develop their career as a GP. This study aimed to investigate negative factors influencing the career intention of GPs in eastern China from the perspective of trainees taking standardized residency training, as to identify the barriers of GP trainees becoming registered GPs, and to provide a policy-making basis for GP recruitment and retention.

**Methods:**

A qualitative description design by the purposive sample was carried out in two training bases of Jinan and Qingdao in eastern China. Face-to-face, in-depth, semi-structured interviews were conducted, audiotaped, and transcribed using thematic analysis.

**Results:**

Twenty-one trainees participated in this study. Thematic analysis generated five major themes: (1) low social recognition, (2) low professional identity, (3) low remuneration level, (4) imperfect training system, and (5) influence of policy factors.

**Conclusions:**

Our results identified various negative factors influencing the career intentions of trainees. In order to overcome the hurdles and increase the attractiveness of GP, it is recommended that the government and the public should create a supportive environment, which can be beneficial to the construction and development of GP.

**Supplementary information:**

The online version contains supplementary material available at 10.1186/s12909-022-03456-x.

## Introduction

General practice (GP) is the backbone of a sound primary care system and those general practitioners (GPs) are the ones to pilot through the health system, who have the potential to play a vital role in quaternary prevention [1] and in reducing health care costs [2]. However, recruitment and retention of GPs has been as a challenge in high-income western countries, such as the UK [3], the USA [4], Germany [5], Canada [6], and elsewhere. And the situation is worse in developing countries [7, 8]. The number of GPs per 100 000 population across China is around 2.9 [9], which is significantly smaller than, for example, that of England(despite having different health systems), which is the 59.5 GPs per 100 000 population [10].

The concept of GP was introduced into China in the late 1980s [11], and GP was formally established as a medical discipline in 1993 [12]. Students in clinical medicine can purse 3- (associate),5- (bachelor), 8- (master) year degrees and above (doctor) [13]. The students with a 3- and 5-year degree can become registered GP assistants and GPs respectively in community health services in the primary care setting, whereas students with an 8-year and above degree can become subspecialists in hospitals. The registered GP, assistant GP, registered nurse, rural doctors, and medics (unlicensed doctors in rural) are the main providers of primary health care services [9].

In 2011, the Chinese government released a policy statement to establish the training system for GPs [14], which can be divided into three major training program categories: post-transfer training program, designated GP undergraduate education program, and residency training program (“5 + 3” model) [15]. The post-transfer training program involves retraining the majority of less-educated doctors(GP assistants), both those who obtained associate degrees who plan to be or have been engaged in community health facilities but are not trained in GP [16]. The designated GP undergraduate education program is to produce qualified GPs for undeveloped areas. The program is 5-year long emphasizes GP and will grant trainees a bachelor’s degree. The training of GP is mainly based on a “5 + 3” model, a 5-year undergraduate medical education (leading to a bachelor’s degree) plus 3 years of standardized residency training (SRT). The 3-year transition period can be carried out in two ways:‘standardized training after graduation’ for residents, which have already practiced clinical medicine with a certificate in medical practice, and ‘postgraduate education of medicine’ for interns [14].

Although more and more medical colleges have started developing GP and the training system of GP has been established in China, the number of GP trainees to be registered GP did not achieve an ideal result. By the end of 2014, the first cohort of SRT resident doctors (totaling 5500) was recruited in 30 Chinese provinces, around 10% of whom (5158) were enrolled as GP residents [17]. One study reported 70.2% (198/282) of the trainees were unwilling to register as GP in a regional survey in China [18]. Another study suggested a relatively or extremely high turnover intention was found in 45.9% (62/135) of trainees in China [19].

Many negative factors can affect trainees in their career intention. Heavy workload, low morale, the loneliness of working in practice, low academic ability, and negative comments about GPs were the most frequently highlighted factors in western countries [20–22]. Limited studies on this issue have been conducted outside the developed western countries. Negative factors influencing trainees’ aspiration to select GP as a career in China can be different from developed countries due to its vastness, diversity, and unique history. Additionally, a few studies focused on burnout and turnover intention of the trainees in SRT in China and were mainly carried out by using questionnaire surveys [18, 19]but they did not reach the core of trainees’ daily practice. So far, only one study adopted a qualitative approach to explore the career intention of trainees [23] and it was conducted in Nanjing city. It is therefore necessary to further explore the factors that hinder trainees to be GPs in China.

This qualitative study aimed to explore the negative factors influencing the career intention of GP trainees in eastern China, as to identify the barriers of GP trainees becoming registered GPs, and to provide a policy-making basis for GP recruitment and retention.

## Method

A qualitative description [24] was used with semi-structured, in-depth interviews to explore negative factors influencing career intention faced by trainees in SRT. This approach allowed personal opinions to be freely expressed which can better capture the thoughts and feelings of trainees’ perspectives to attain our research aim.

### Setting/participants

Training bases are medical and health institutions that undertake standardized training for resident physicians, most of which are located in tertiary hospitals with sufficient talents and medical resources [25]. The mentors in the training base are specialists, who undertake the teaching task of GP on a part-time basis. The study was conducted through two training bases in Jinan and Qingdao, which hold vast GPs resources in Shandong Province [26].

### Participants and recruitment

We used purposive sampling for trainees. Trainees in SRT were recruited from two training bases in two cities of eastern China (Jinan and Qingdao), with the help of hospital education managers. The hospital education managers were served as a liaison between trainees and researchers. They first introduced the study purpose to trainees who were potentially eligible to participate in the study. They then introduced the researchers to trainees, so the researchers built a relationship of trust with the trainees. The researchers then contacted those trainees who were interested and who met inclusion criteria in participating interviews and made an appointment to meet at a convenient time and place. The inclusion criteria were: (1) GP trainees belonging to the “5 + 3” training model who was willing to participate in the study, (2) who had been taking standardized training courses for at least one year. We attempted to reach maximal variation in sampling by recruiting trainees from a wide range of backgrounds, including age, gender, years of training, and marital status. Trainees’ demographic information is presented in Table 1.

**Table 1:** Trainees’ characteristics (*N* = 21)

Characteristics of trainees	Descriptions	n (%)
Age range	20–29	17 (80.9)
	30–39	4 (19.1)
gender	Female	15 (71.4)
	Male	6 (28.6)
Training base	Jinan	11 (52.4)
	Qingdao	10 (47.6)
Years of training	1	7 (33.3)
	2	11 (52.4)
	3	3 (14.3)
marital status	Yes	8 (38.1)
	No	13 (61.9)

### Data collection

Face-to-face semi-structured interviews were conducted in Mandarin Chinese by research assistants trained in qualitative methods from June to August 2019. Before each interview, the purpose of the interview was explained, and consent to record the interview on audiotape was confirmed by all trainees. To create a relaxed atmosphere for communication, interviews were usually carried out in the lounge or intern office. Generally, there were only two people (a trainee and the researcher) in the room, to better encourage trainees to express themselves truthfully.

The first step of our study was to prepare a list of core questions. The core questions of this study were developed based on: (1) The *Guidelines of The State Council on the Establishment of a General Practitioner System* on the official website of National Health Commission of the People’s Republic of China;(2) a review of the existing literature on what influences trainees’ career intention. We conducted two pilot interviews when trainees were asked to respond to the prepared open questions such as ----“Tell me about your career intention in the future”, and to illustrate the reasons for their decision. No substantial changes were needed after piloting with two trainees. A more detailed interview guide is presented in Additional file 1. Semi-structured interviews allowed trainees to freely share their thoughts and perceptions but allowed the researchers to retain some control over the direction of the study. The researchers used interview techniques to encourage the participants via verbal (e.g. “your answers were quite illuminating”) or non-verbal (e.g. establishing eye contacts) incentives.

The interviews lasted 30 to 60 mins. Field notes were taken during the interviews to capture non-verbal data. The length of the interview varied according to the stage of saturation and the emergence of new themes. The researchers continued interviewing new trainees until no new themes emerged. In the end, a number of 21 interviews were conducted.

### Data analysis

Interviews were transcribed verbatim within 24 h after the end of each interview. The transcribed material was thoroughly checked by the interviewers to ensure that the audios were correctly transcribed. To ensure that the transcripts truly reflect what the trainees said, the researcher returned the transcripts to the trainees for another round of data checking. Data were analyzed using an inductive thematic analysis [27], in which coded categories were derived directly from the transcripts. To ensure consistency, researchers strictly followed the steps of the analysis process [28]. First, data for each of the trainees were read several times, and their perceptions, experience, and intention about being GP were identified and carefully listed. Then, segments of transcripts were categorized according to the salient theme of the segment by two experienced researchers, a process called “coding”. They met regularly (about after every 4–5 interviews) to discuss and validate codes. Any inconsistencies in coding assignments were resolved in discussions. The second step of data analysis mainly focused on the deduction and induction of coding by comparison and integrated into a theme. Capturing salient patterns of data and explanations for the stated perceptions and experiences provided by the trainees were emphasized in this process. In a constant comparison process, the ascribed codes were compared with new data from the subsequent interviews and adapted if necessary. In the third step, researchers checked to make sure no information was missing and reached a consensus on the final categories. Finally, categories were translated and reported in English.

## Results

### Sociodemographic characteristics of participants

There was no refusal of consent or dropout during interviews, with a total of 21 trainees participating. Of these, the age of the trainees ranged from 23 to 36 years (median age = 28 years); fifteen were female; seven had experience of a GP placement for one year, eleven for two years, and three for three years (mean = 1.9 years); eight were married and the rest among them were single.

### Key themes

The study presented five overarching themes that described negative factors influencing the career intention of the trainees in SRT: low social recognition, low professional identity, low remuneration level, imperfect teaching system, and influence of policy factors.

#### Low social recognition

##### Little knowledge

It was a common phenomenon that the term “general practitioner” was not usually understood by the public in China. Most of the trainees did not know about GP until they joined the training, and said that had little knowledge and contact with GPs before. They stated that GP received little recognition and acknowledgment from the public. There was little recognition even from senior colleagues and peers in the hospital.

*"I am a doctor of this hospital where I’m also a trainee. When I go to other departments for rotation, I find almost none of them know what GP is. They would ask what GP do, and lots of my friends also ask me this question." *(02, Male, 36years)

*"People think it doesn’t really matter if there’re no GP, or, the recognition of GP is really low, even not well among doctors." *(01, Female, 31years)

##### Negative impression of GP

There was also a negative impression about GP among the public, as some of the trainees illustrated how family or friends portrayed GPs.

*"My family and friends know very little (about GPs). They’d say “those are the doctors in town health centers, isn’t it right? You really would work there?” Some of my schoolmates working in county hospitals would say “that’s where you would go? You might as well come home and work at this county hospital.” *(18, Female,24 years)

##### Patients distrust

Chinese patients normally held a concept that a hospital in a higher tier and with a higher level of specialization tends to offer better quality of health care services. Most of the trainees indicated that patients doubted GP's ability to provide medical care and tended to favor specialists in secondary or tertiary hospitals.

*"Patients’ acceptance is still not well. When we take the training, if we tell patients we’re GPs, they’d question our ability." *(05, Male,29 years)

#### Low professional identity

##### Unrecognized value

Although GP trainees should be regarded as those who tend to take a more holistic approach than their specialist counterparts, in our interviews, some of the trainees judged themselves as less competent than other specialties. They tended to have negative opinions regarding the professionality and competence of GP. They expressed that there was less value out in the work of GP.

*"I don’t think GP can meet the primary needs. Even chronical diseases require deep understanding of each symptom, which I don’t have. I can’t give them specific instructions. They still need specialists." *(12, Female,28years)

##### Limited career development

Some trainees felt it would be a waste of their medical degree since there was a lack of clinical work content and there was little room for career development at the grassroots level for GP, compared to hospital medicine.

*"If I work at grassroots level, I would probably be doing some clerical work such as making health records. I wouldn’t like it if my job is not clinic related. All these years of study and 3 years of training would go to waste if I were not a doctor." *(14, Female,30years)

##### Concern of the prospect

The prospect of GP also caused concern for most of the trainees, which seemed to drive trainees away from primary care.

*"From higher levels to grassroots level, many big hospitals might have already understood the importance of GP and established GP departments, but there’s still a long way to go for hospital of lower levels. It’s impossible for patients to acknowledge this GP department within a couple of years." *(01, Female, 31years)

#### Low remuneration level

##### Guaranteed minimum remuneration

Trainees expected to earn a guaranteed minimum remuneration since the salary was considered to be a fair return on trainees’ years spent in medical education and a way to make their lives secure. However, most of the trainees from our interviews expressed that they were unsatisfied with the current remuneration of GPs. Low remuneration level appeared to be a primary driver of intention to not choose GPs.

*"Doctors are people too, and have family to support. Medical students work very hard to go through all these years, 5 years of undergraduate study and 3 years of training. It’s not easy." *(11, Male,28years)

*"The least is to support life. That’s the first point. You have to meet all the living needs before you can focus on your job. This is the most basic." *(13, Female,26years)

#### Imperfect training systems

##### Unprofessional supervisor

Some supervisors appeared to design their teaching syllabus based on their preference and sometimes used fixed syllabus, rather than responding to the needs of the trainees.

*"My supervisor doesn’t care which discipline we’re from. When she takes us on ward rounds, she teaches us something based on what diseases we see, not on each trainee’s discipline." *(09, Female, 29years)

##### Unreasonable training programs

In addition, the curriculum of GP training did not highlight the characteristics of GP. Most the trainees thought there was a lack of outpatient experience in their training.

*"I think the curriculum needs to be improved. When we take the training, we spend most time doing ward rounds. But at grassroots levels, most work we do would be at the outpatient clinic. And admitted patients at a tertiary hospital could have rather severe symptoms, which are rare at grassroots levels." *(10, Male, 30years)

#### Influence of policy factors

##### Incomplete supporting policies

Some the trainees argued that a series of improved medical policies must be in place. Trainees stated that the first-visit care system and the two-way referral system between GPs and specialists should help guide patients and for reducing the waste of resources; yet, such systems were too weak in China.

*"(The process should be that) an almost recovered patent of a higher level hospital should go to a lower level hospital for further recovery, and a patient from a lower level hospital should go to a higher level hospital for treatment if requested. But now many policies are defective." *(10, Male, 30years)

##### Poor implementation of policies

A few of the trainees expressed frustration at the implementation of the policy because there were gaps between what was called for and what was actually done.

*"I think more efforts should be made on implementation. Otherwise they’re all empty promises." *(03, Female, 28years)

## Discussion

This study explored negative factors influencing the career intention of GP trainees in China. Despite significant improvements have been made in general medicine education, these negative factors influencing trainees’ career intention show there is still plenty of room to improve GPs recruitment.

According to our study, GP received little recognition and acknowledgment from the public. On the one hand, GP is a relatively new clinical discipline in China, which has not formed a professional status and discipline field [29]. The public and even trainees’ peers had not embraced the concept of GP and are not accustomed to community health care services. This is different from western countries, where GP has become a common aspect of their medical system [30]. On the other hand, the inadequate use of primary health care is also contributed to GPs’ low social acceptance. Patients consider that those receiving 3–5year medical education cannot meet their needs, which leads patients skipping the community health centers and going straight to the outpatient departments of tertiary hospitals [31].

The Chinese health care system emphasized the importance of urban hospitals with specialized departments as well as the struggle against specific diseases. The role of GPs in community health centers or rural are often responsible for “minor ailments” or chronic diseases in the curative realm, thus GPs seem to be appreciated as an ‘easy’ medical specialty in China. Previous studies, however, showed that the professional identity level among GPs in China was high [32, 33]. One possible interpretation could be that compared with GPs, trainees in our study had less work experience and had less opportunity to understand the mission of GPs. Serval studies highlighted the important influence of the workplace experience and the length of train time on recruitment [34, 35]. The extensive work experience of GP means more clinical exposure. Evidence showed that the quantity of clinical exposure helped promote a number of characteristics, including the acquisition of knowledge and skills, and encourage them to take on more complex clinical responsibilities [36].Our interpretation was supported by these studies.

The pay for GPs was low, and central funds were scarce for them in China. The remuneration structure of GPs in China can be divided into two parts: basic performance and incentive performance. To reduce the phenomenon of over-prescription, the Chinese government introduced a zero-mark-up drug policy in 2009 [37]. However, the removal of drug mark-up has in turn affected the remuneration of GPs at the grassroots, as the GP’s bonuses depend on drug revenues [38]. So far there were no effective measures to compensate for these income losses. Studies showed that Chinese GPs who worked in primary health care (PHC) institutions usually received lower income than specialists in secondary/tertiary hospitals whose remuneration flatted with the social average wage (1 time or so) [39, 40]. Also, GPs do not receive legally mandated social benefits. A review reported that employees in most of community centers, community health stations, and township health centers had no social benefits mandated by the Chinese Government [41]. This reflects the problem of inadequate financial support.

Unsatisfying training system was an important negative factor that emerged in this study, actually, other Western countries such as Germany also struggle hard to improve the training of GP and improve its attractiveness [42]. In the current Chinese context, most GP teaching physicians are hospital-based specialists, and they have little contact with GPs themselves [43]. GPs trainees thus do not well understand the complementary aspects between GP and specialist, if their supervisors do not give appropriate and adequate instruction. A previous study reported that infrastructure and clinical care capabilities provided by primary healthcare institutions would not satisfy patients’ needs, which can diminish their preparedness to care for the public [41]. This could also be one of the reasons the GPs profession was not as attractive, but it was not well explored in our study.

Shortage of GPs, poor service skills, and low social recognition and professional identity are the main limiting conditions influencing the development of GPs responsibility system and two-way system [44]. These flawed systems, in turn, impede the trainee’s decision to become a GP. Developing a system to make GPs the first option is necessary for promoting GP [45]. The other challenge is the lack of recognition of the importance of the reform in the policy. The long-term benefits of the GP reform are not well understood by bureaucrats of local government and staff in the district hospitals, who find it difficult to implement policies [46]. Strengthening leadership and organizational structure are required for GP’s career development in the future.

### Strengths and limitations of this study

Trainees with different demographic characteristics, including genders, training years and experience, contributed to this study, which helped provide a diverse and complete profile of GP trainees. The limitations of our study are as follow. First, the transferability of our findings can be limited, since the findings were obtained with in the special context of China, where the GP system has just been established; although the Chinese reform systemic strategics and problems can become an exemplary for other nations that would develop GPs training, such as Pakistan [47]. Additionally, although we attempted to maintain a position of neutrality throughout the data collection and analysis, it was nevertheless possible that certain trainees may be reluctant to express their authentic views but give the researcher’s desired responses instead. To minimize this potential bias, we actively encouraged the trainees to express their views and always challenged dominant viewpoints, for example, by asking ‘does anyone have any different opinions on this subject?’

## Conclusions

We have identified negative aspects which can influence the career intention of trainees, including social recognition, professional identification, remuneration, training system, and policy. These results suggest that more work needs to be done to increase the attraction of GP. It is hoped that through educational campaigns, the importance of community health services and the status of GPs can receive more recognition from the public. Additionally, the government should constantly strengthen policy support, such as a fair remuneration system, evaluation system of training curriculum, and management system of GP trainers. It is also necessary to strengthen leadership and organizational structures to ensure policy implementation to attract highly qualified GPs to provide primary care in China.

## Supplementary information

**Additional file 1.** The interview outline of negative factorsinfluencing career intention: a qualitative study of the general practicetrainees.

## Data Availability

The data used and/or analyses during the study are available from the corresponding author upon reasonable request.
