# Supplementary material for: The negative factors influencing the career intention of general practice trainees in eastern China: a qualitative study
Source: BMC Med Educ. 2022 May 21;22:393. doi: 10.1186/s12909-022-03456-x (PMC9124415; doi:10.1186/s12909-022-03456-x)
Supplement: Supplementary file 1 — Additional file 1. The interview outline of negative factorsinfluencing career intention: a qualitative study of the general practicetrainees. [file 12909_2022_3456_MOESM1_ESM.docx]

**Additional file 1: The interview outline of negative factors influencing career intention: a qualitative study of the general practice trainees**

**Before the interview:**

- Thank you for participating in our survey.
- The aim of this qualitative study was to explore negative factors influencing trainees’ career intention, providing a policy-making basis for GPs recruitment and retention. All your personal information will be kept strictly confidential at all times. All investigation information will only be used for academic research and will not be disclosed.
- Our interview will last 60 min, and you are free to terminate the interview at any time.
- Do you mind if I record it by audio recorder in order to accurately convey what you have told me? (If they have agreed to allow recording of the interview then start recording now, otherwise take notes.)

**Collection of basic information**

(Interviewees are asked to fill out a basic information form, again emphasizing confidentiality)

**Summarize the interview outline about the trainees' career intention**

- ***Attitudes of general practice/GPs***

1. Please talk about your views on GP. Why do you think so?
2. Have you ever heard what else people think about GP? If so, what did they think?
3. Why did you choose GP as your discipline?

4. What do you think of the development of GP/GPS?

- ***Factors influencing career intention***

1. Tell me about your career intention in the future. why?
2. Please talk about the barriers registering to be a GP.
3. If there are other career opportunities after the program, would you choose to leave your major? What's the reason?

- ***Suggestions for improving attractive of GPs***

1. Based on the current situation of GP, what do you think is the urgent need for the development of GP?
2. What are your suggestions on how to enhance the attractiveness of GPs?

**End of interview**

Thank you for your time and contribution to this study.
